# Chinese Facial Beauty Preference

**DOI:** 10.1007/s00266-018-1228-5

**Published:** 2018-10-16

**Authors:** Xingchen Zhu, Xiao Long

**Affiliations:** 1 Communication University of China, Beijing, China; 2 Division of Plastic Surgery, Peking Union Medical College Hospital, Chinese Academy of Medical Sciences and Peking Union Medical College, No. 1 Shuaifuyuan, Wangfujing, Dongcheng District, Beijing, 100730 China

*Level of Evidence V* This journal requires that authors assign a level of evidence to each article. For a full description of these Evidence-Based Medicine ratings, please refer to the Table of Contents or the online Instructions to Authors www.springer.com/00266.

Dear Sir,

With great interest, we read the article ‘Ideals of Facial Beauty Amongst the Chinese Population: Results from a Large National Survey’ written by Dr. Souphiyeh Samizadeh and Prof. Woffles Wu [1]. In the paper, the authors investigate ‘the preference of Han Chinese laypersons for facial shape, profile (straight, convex, concave), jaw angle and shape, and shape of the chin, nose, and lips’ [1]. From this survey, the authors concluded that the Han Chinese population prefers an oval facial shape with minor variations to the oval facial shape, a pointed, narrow chin, obtuse mandibular angle for women and a straight facial profile and to some extent an anteriorly projecting chin, a concave or straight dorsum of the nose and small, full lips with well-defined Cupid’s bows with tapering volume towards the oral commissures.

Recently, we have carried out a similar investigation to gain the Chinese aesthetic preference for the angles and proportions of midface through three-dimensional facial images processed by Photoshop CS6. We took a three-dimensional facial image of a 25-year-old Chinese woman, who had no plastic surgery history and no facial deformity. Midface angles and proportions including nasofrontal angle, nasolabial angle, intercanthal width/nose width and ocular width/mouse width were modified to various values by Photoshop CS6 [2]. A total of 1333 Chinese raters (including 338 plastic surgery patients and 995 people with no plastic history) were recruited to offer their own demographic information such as gender, age, educational background, economic condition and plastic history, and to choose the most ideal angle or proportion. In the preliminary results, we found the Chinese ideal nasofrontal angle, nasolabial angle, intercanthal width/nose width and ocular width/mouth width were 133.99° ± 5.49°, 89.65° ± 5.55°, 0.84 ± 0.07 and 0.64 ± 0.03. Women in North China preferred a larger nasolabial angle, smaller nasofrontal angle and larger ocular width/mouth width than those in South China. Elderly Chinese with low education levels, high income and high expense had an obvious preference for a small intercanthal width/nose width.

**Fig. 1:**
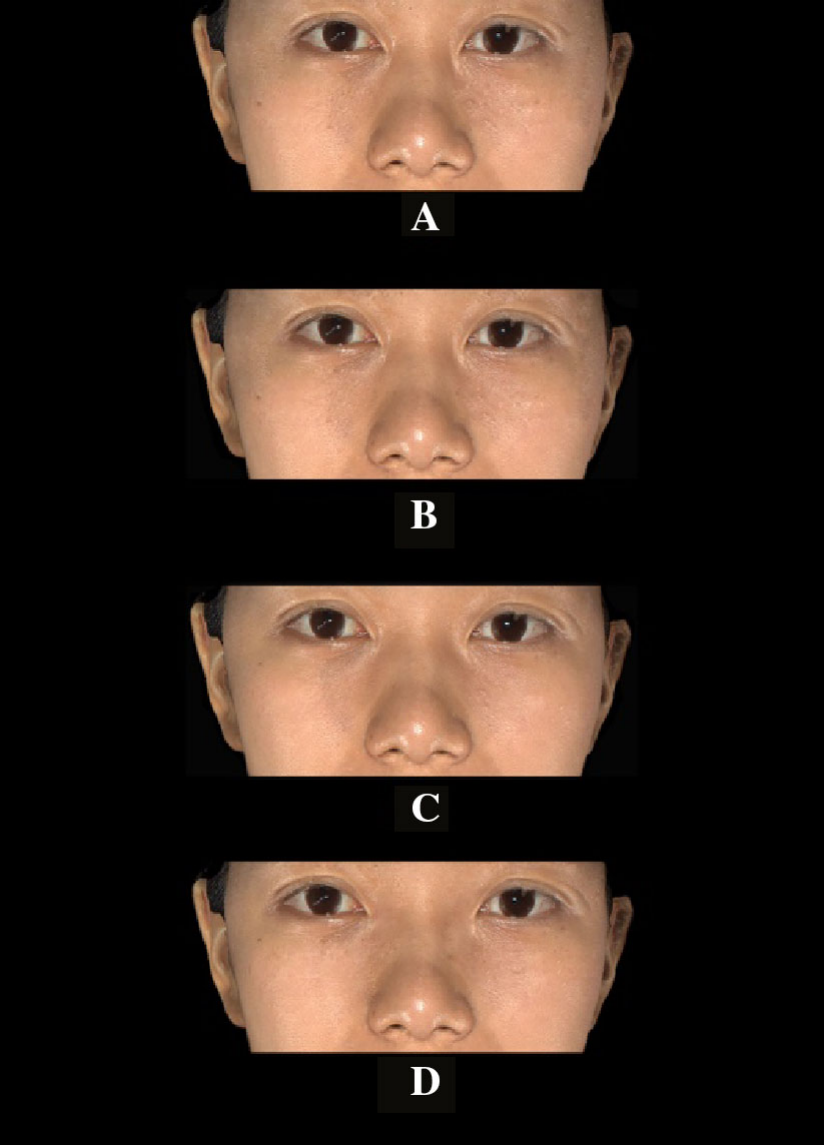
Investigation picture of different intercanthal widths

We think our investigation results could be the supplement data for the Samizadeh and Wu study. By combining the methods and the results, plastic surgeons from different areas of Asia could have a more thorough picture of Chinese beauty and the Chinese aesthetic characteristics could be concluded as well (Fig. 1).
